# Astragalin Attenuates Dextran Sulfate Sodium (DSS)-Induced Acute Experimental Colitis by Alleviating Gut Microbiota Dysbiosis and Inhibiting NF-κB Activation in Mice

**DOI:** 10.3389/fimmu.2020.02058

**Published:** 2020-09-15

**Authors:** Lei Peng, Xiaoyu Gao, Long Nie, Jing Xie, Tianyi Dai, Chongying Shi, Liang Tao, Yan Wang, Yang Tian, Jun Sheng

**Affiliations:** ^1^Engineering Research Center of Development and Utilization of Food and Drug Homologous Resources, Ministry of Education, Yunnan Agricultural University, Kunming, China; ^2^College of Food Science and Technology, Yunnan Agricultural University, Kunming, China; ^3^Yunnan Province Research Institute of Plateau Characteristic Agricultural Industry, Kunming, China; ^4^Yunnan Provincial Key Laboratory of Biological Big Data, Yunnan Agricultural University, Kunming, China; ^5^Yunnan Provincial Engineering Research Center for Edible and Medicinal Homologous Functional Food, Yunnan Agricultural University, Kunming, China; ^6^Key Laboratory of Pu-er Tea Science, Ministry of Education, Yunnan Agricultural University, Kunming, China

**Keywords:** Astragalin, *Escherichia-Shigella*, gut microbiota, NF-κB, Ruminococcaceae, ulcerative colitis

## Abstract

With the ulcerative colitis (UC) incidence increasing worldwide, it is of great importance to prevent and treat UC. However, efficient treatment options for UC are relatively limited. Due to the potentially serious adverse effects of existing drugs, there is an increasing demand for alternative candidate resources derived from natural and functional foods. Astragalin (AG) is a type of anti-inflammatory flavonoid, with *Moringa oleifera* and *Cassia alata* being its main sources. In this study, we investigated the therapeutic effects of AG on mice with dextran sulfate sodium (DSS)-induced colitis. Our results suggested that AG treatment reduced weight loss and the disease activity index (DAI), prevented colon shortening and alleviated colonic tissue damage. AG treatment reduced the expression of pro-inflammatory cytokines and related mRNAs (such as *TNF-*α*, IL-6*, and *IL-1*β), inhibited colonic infiltration by macrophages and neutrophils, ameliorated metabolic endotoxemia, and improved intestinal mucosal barrier function (increased expression levels of mRNAs such as *ZO-1, occludin*, and *Muc2*). Western blot analysis revealed that AG downregulated the NF-κB signaling pathway. Moreover, AG treatment partially reversed the alterations in the gut microbiota in colitis mice, mainly by increasing the abundance of potentially beneficial bacteria (such as Ruminococcaceae) and decreasing the abundance of potentially harmful bacteria (such as *Escherichia-Shigella*). Ruminococcaceae and Enterobacteriaceae (*Escherichia-Shigella*) were thought to be the key groups affected by AG to improve UC. Therefore, AG might exert a good anti-UC effect through microbiota/LPS/TLR4/NF-kB-related pathways in mice. The results of this study reveal the anti-inflammatory effect and mechanism of AG and provide an important reference for studying the mechanisms of natural flavonoids involved in preventing inflammation-driven diseases.

## Introduction

Ulcerative colitis (UC) is one of the main types of inflammatory bowel disease (IBD) and has an increasing incidence worldwide. Its pathogenesis is multifactorial, including genetic susceptibility, epithelial barrier defects, changes in the intestinal microbiota, immune dysregulation, and environmental factors (1, 2).

In recent years, the intestinal microbiota has been widely recognized to regulate intestinal homeostasis and the pathogenesis of UC. Changes and disorder in the gut microbiota are closely related to the occurrence or development of UC, but whether disorder is primary or secondary is unclear (3). The mechanisms involved are still not fully understood.

Currently, 5-aminosalicylate (5-ASA), glucocorticosteroids, azathioprine and cyclosporine are commonly used drugs for UC treatment (4). Unfortunately, the long-term use of these drugs has been found to cause severe toxicity; therefore, it is increasingly necessary to explore alternative candidate agents derived from natural resources and functional foods for UC.

Astragalin (AG), a natural flavonoid, has been found in various traditional medicinal and edible plants, such as *Moringa oleifera, Radix astragali, Morus alba*, and *Cassia alata*. AG exhibits various pharmacological properties, including anti-inflammatory, antioxidant, neurological, cardioprotective, antidiabetic, and anticancer effects (5). Previous studies on the anti-inflammatory activity of AG in animals have mainly reported the effects of AG on mastitis, endotoxemia, lung injury, allergic inflammation and hepatic fibrosis in mice or rats (5). Astragalin has been reported to modulate inflammatory responses by regulating the expression of NF-κB, iNOS, cytokines/chemokines, MAPK signaling pathway components, and PAR2 signaling pathway components (6). However, to date, there have been no reports on the treatment effects of AG on UC and the gut microbiota. Most flavonoids or polyphenols have low bioavailability and a low absorption rate, and up to 90% of these compounds remain in the colon (7). Polyphenols such as resveratrol, epigallocatechin-3-gallate, curcumin, quercetin, and anthocyanin have been verified to have therapeutic roles in IBD animal models (8–12). Their therapeutic functions in IBD usually include regulating the NF-κB pathway and gut barrier function, and a large number of studies have shown that the gut microbiota is also an important target (13). This finding provides a theoretical basis for the hypothesis that AG and its metabolites may alleviate UC by regulating the gut microbiota.

Here, we evaluated the anti-UC activity of AG in a dextran sodium sulfate (DSS)-induced UC mouse model. Ultimately, the anti-UC effects and mechanisms of AG related to anti-inflammatory activity, NF-κB signaling pathway downregulation, intestinal barrier improvement, and gut microbiota regulation were evaluated.

## Materials and Methods

### Reagents

AG (purity > 98%) was purchased from Chengdu Derick Biotechnology Co., Ltd. (Chengdu, China). AG is difficult to dissolve in water (or PBS), so we dissolved it in DMSO. DSS (36,000 to 50,000 Da) was purchased from MP Biomedicals (Solon, OH, USA). 5-ASA and RIPA cell lysis buffer containing 1% phenylmethanesulfonyl fluoride (PMSF) were obtained from Solarbio (Beijing, China). A myeloperoxidase (MPO) detection kit was obtained from Nanjing Jiancheng Bioengineering Institute. Antibodies against β-actin (#4970), IκBα (#4812), phospho-IκBα (#4792), p65 (#5970), phospho-p65 (#3031), IKKα (#61294), IKKβ (#8943), and p-IKKα/β (#2697) were purchased from CST (Boston, MA, USA). An anti-rabbit IgG antibody was purchased from R&D Systems (Minneapolis, MN, USA).

### Animal Experimental Design

The experimental protocols were approved by the Yunnan Agricultural University Animal Ethics Committee with respect to ethical issues and scientific care (YNAU-2018-031).

**Figure 1:**
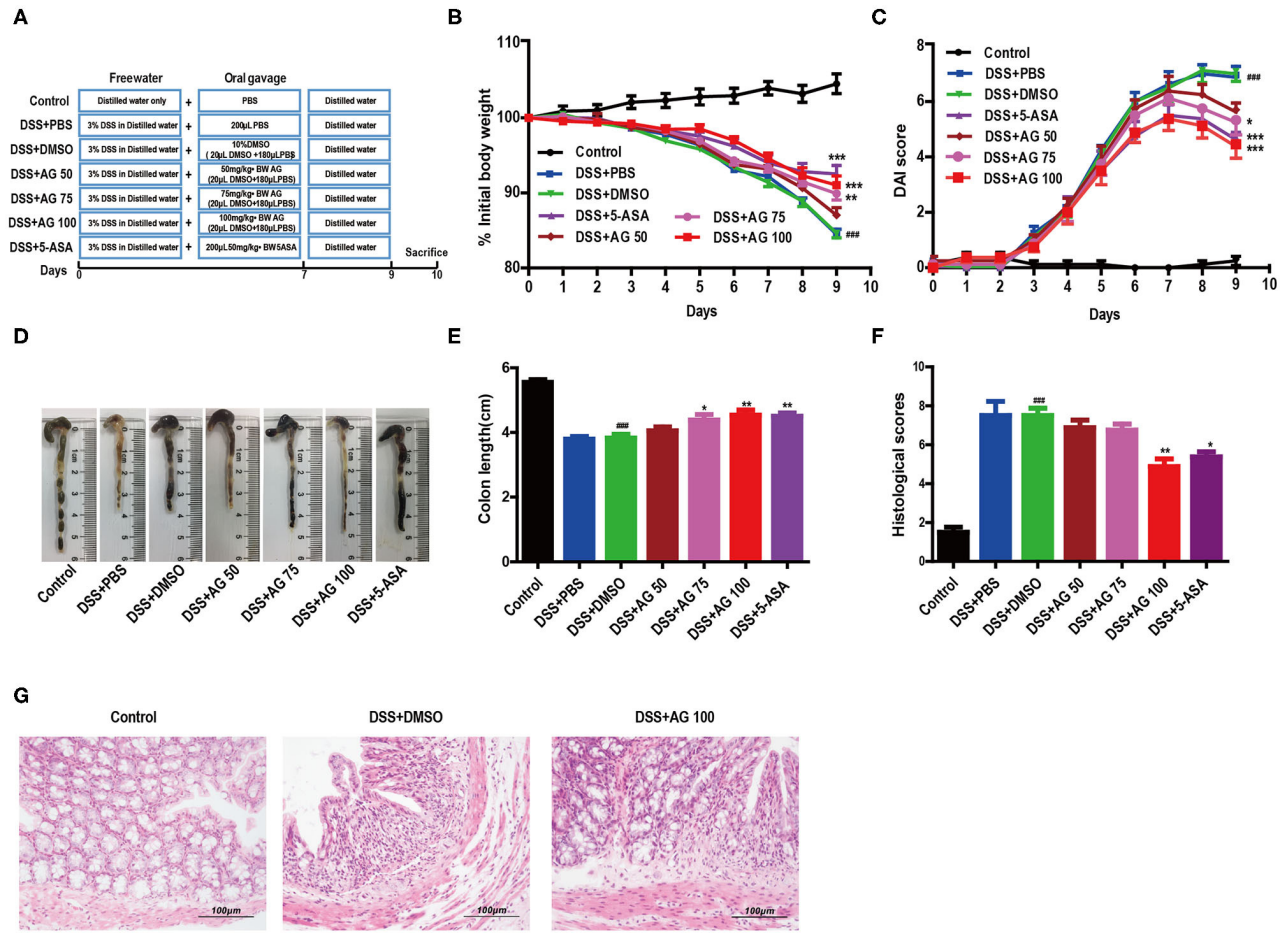
AG attenuates symptoms of DSS-induced colitis in mice. **(A)** Schematic diagram of the animal experimental design. **(B)** Mouse body weights measured daily. **(C)** Calculated DAI scores. **(D,E)** Intestine images and statistics for colon length in each group. **(F)** Histological scores of colonic tissue. **(G)** Representative HandE-stained images of colon sections (400 × magnification). Data are expressed as the mean ± SEM, *n* = 8. Data were analyzed using one-way ANOVA (*post hoc* Tukey's multiple-comparison test). ^###^*P* < 0.001 compared with the control group. *P < 0.05, **P < 0.01, and ***P < 0.001 compared with the DSS + DMSO group.

Male C57BL/6J mice (6 weeks old, 17-21 grams, *n* = 56) were purchased from the Model Animal Research Center of Nanjing University (Nanjing, China) and housed under constant conditions (room temperature 24 ± 1°C, 12 h light/dark cycle, lights off at 20:00) with clean water and the AIN-93M diet (Trophic Animal Feed High-tech Co., Ltd., Nantong, China) available *ad libitum*. After 1 week of acclimation, the mice were randomly divided into seven groups of 8 mice each according to their body weights as follows: a control group (received water as vehicle); DSS + PBS group; DSS + DMSO group; DSS + 50 mg/kg AG group (DSS + AG50); DSS + 75 mg/kg AG group (DSS + AG75); DSS + 100 mg/kg AG group (DSS + AG100); and DSS + 50 mg/kg 5-ASA group (DSS + 5-ASA). AG was dissolved in DMSO. The DSS + DMSO group was the solvent control. The DSS + PBS group was set up to exclude any unknown effects of the solvent DMSO on the experimental results. A DSS-induced UC mouse model was generated as previously described (14). Briefly, the colitis was induced by administration of 3% DSS for 7 days, and at the same time, the mice were gavaged with daily oral doses of 200 μL of different solutions. Different doses of AG were dissolved in 20 μL of DMSO + 180 μL of PBS. All the mice were freely allowed normal water for 2 more days to observe the development of colitis (Figure 1A).

Body weight changes and the disease activity index (DAI) were recorded during the entire animal experiment. The DAI is the sum of scores for weight loss, stool consistency, and the gross bleeding extent. The detailed scoring criteria for the DAI (15) are described in Table S1. After 12 h of fasting, the mice were sacrificed by CO_2_ asphyxiation at the end of the experimental period.

### Histological Assessment

Colon tissue samples were fixed in 10% formalin and then embedded in paraffin. All tissue samples were cut into 3 μm sections for staining with hematoxylin and eosin (H&E). All H&E-stained tissue sections were assessed using the following scoring scheme (16), including inflammatory infiltration (0–5), crypt injury (0–4), ulcer (0–3), and presence or absence of edema (0 or 1). All histological scoring was performed by 2 blinded investigators.

### Immunohistochemistry and an Immunoblotting Assay

Immunohistochemistry was performed using a previously described method (17). Briefly, 3 μm sections were deparaffinized in xylene and rehydrated in graded alcohol. After quenching endogenous peroxidase activity and blocking non-specific binding, the sections were incubated with a mouse monoclonal antibody against CD177 or F4/80 (Santa Cruz, CA, USA) overnight at 4°C and then incubated with a biotinylated secondary antibody (Zymed Laboratories, Carlsbad, CA, USA) at room temperature for 20 min. Finally, the slides were incubated with reagents from the Avidin-Biotin Complex Kit (Vector Laboratories, Burlingame, CA, USA) and a 3,3'-diaminobenzidine kit (Tiangen, Sichuan, China) according to the manufacturer's instructions. Images were captured with an Olympus CX43 microscope and CellSens Entry software.

### Real-Time PCR Analysis

Total RNA was extracted from colonic samples using TRIzol (TransGen Biotech, Beijing, China) according to the manufacturer's protocol and quantified using a NanoDrop 2000 Spectrophotometer (Thermo Fisher Scientific Inc., Waltham, MA, USA). Complementary DNA was synthesized using a PrimeScript RT reagent kit with genomic DNA eraser (Takara, Dalian, China) in accordance with the manufacturer's protocol. Quantitative real-time polymerase chain reaction (RT-qPCR) was performed with SYBR® Premix Ex Taq™II (TliRNaseH Plus, Takara, Dalian, China) and the ABI 7900HT Fast Real-Time PCR System (Applied Biosystems, Inc.). The relative amount of the target mRNA was normalized to the β*-actin* level, and results were calculated by the 2^−ΔΔ*Ct*^ method. The specific primers used are indicated in Table S2.

### Enzyme-Linked Immunosorbent Assay (ELISA)

Colon tissue samples were homogenized in a buffer containing protease inhibitors for 1 min. The mixture was incubated on ice for 15 min and then centrifuged at 15,000 g for 10 min to obtain the supernatant. The concentrations of monocyte chemoattractant protein 1 (MCP-1), tumor necrosis factor alpha (TNF-α), IL-1 beta (IL-1β), and interleukin 6 (IL-6) in the supernatants of the colon tissue samples were measured using ELISA kits (Beijing 4A Biotech Co., Ltd., Beijing, China) according to the protocol of the manufacturer. A Cusabio ELISA kit (Wuhan, China) was used to determine the serum LPS concentration.

### MPO Activity

Colon tissue samples from approximately the same site were weighed and homogenized in ice-cold PBS. MPO activity was measured using a kit according to the manufacturer's instructions. MPO activity was measured using the O-dianisidine method and is reported as units per gram of wet tissue.

### Western Blot Analysis

Colon tissue samples were minced and placed in Eppendorf tubes. RIPA buffer (Solarbio Life Science, Beijing, China) supplemented with a protease and 1% PMSF was used to isolate total protein from the colon tissue samples. A BCA protein kit (Beyotime Biotechnology, China) was used to determine the protein concentration. Whole amounts of protein (60 μg) were loaded onto a 10% sodium dodecyl sulfate polyacrylamide gel. Membranes were blocked with 5% bovine serum albumin after the proteins were transferred to PVDF membranes (Millipore, MA, USA). Then, the membranes were incubated overnight at 4°C with the following primary antibodies: anti-β-actin, anti-IκBα, anti-p-IκBα, anti-IKKα, anti-IKKβ, anti-p-IKKα/β, anti-p65, and anti-p-p65. The next day, the membranes were washed with PBST buffer and incubated with appropriately diluted horseradish peroxidase-conjugated secondary antibodies for 1 h at room temperature. After the membranes were washed again 3 times, the protein bands were visualized using an enhanced chemiluminescence kit (Tiangen Biotech, Beijing, China) according to the manufacturer's instructions.

### Sequencing of the 16S rRNA Genes of the Gut Microbiota and Bioinformatic Analysis

Collected mouse cecal contents were stored at −80°C after being snap frozen in liquid nitrogen. The cecal content samples were sent to Majorbio Biotechnology Co., Ltd. (Shanghai, China) under dry ice conditions for 16S rRNA gene sequencing. Microbial DNA was extracted from the mouse cecal contents of the control group, the DSS + DMSO group and the DSS + AG 100 group using the E.Z.N.A.® soil DNA Kit (Omega Bio-tek, Norcross, GA, USA) according to the manufacturer's protocols. The final DNA concentration and purity were determined with a NanoDrop 2000 UV-Vis spectrophotometer (Thermo Scientific, Wilmington, USA), and DNA quality was checked by 1% agarose gel electrophoresis. The V3-V4 hypervariable regions of the bacterial 16S rRNA gene were amplified with the primers 338F (5′- ACTCCTACGGGAGGCAGCAG-3′) and 806R (5′-GGACTACHVGGGTWTCTAAT-3′) using a thermocycler PCR system (GeneAmp 9700, ABI, USA). Detailed information on the PCR procedures, purification and quantification methods of the PCR products are described in the online Supplementary Materials.

Purified amplicons were pooled in equimolar amounts and paired-end sequenced (2 × 300) on an Illumina MiSeq platform (Illumina, San Diego, CA, USA). The taxonomy of each 16S rRNA gene sequence was analyzed by the RDP Classifier algorithm against the Silva (SSU123) 16S rRNA database using a confidence threshold of 70%. Subsequent bioinformatic analysis was performed on the cloud platform of Majorbio Bio-Pharm Technology Co., Ltd. (Shanghai, China).

### Statistical Analysis

Data are presented as the arithmetic mean ± standard error of the mean (SEM). Data were analyzed using GraphPad Prism 5.0 (GraphPad, San Diego, CA, USA). Statistically significant differences between groups were evaluated by one-way analysis of variance (ANOVA) followed by Tukey's honest significant difference test. Bivariate correlations were calculated using Pearson *r* coefficients. HemI 1.0 software was used to construct heatmaps.

## Results

### AG Inhibited DSS-Induced Colitis Symptoms

A schematic of the animal experimental design is shown in Figure 1A. The body weight of the mice in the DSS + PBS group and the DSS + DMSO group decreased throughout the animal experiment, while the body weight of the mice in the control group increased. The inhibition of weight loss in the DSS + AG groups (75 mg/kg and 100 mg/kg) was comparable to that observed in the DSS + 5-ASA (50 mg/kg, positive drug control) group (Figure 1B).

The DAI score increased significantly after DSS intake, whereas it was markedly attenuated in the DSS + AG groups (75 mg/kg and 100 mg/kg) and the DSS + 5-ASA group (Figure 1C). Moreover, our results indicated a significant shortening of the colon in the DSS + DMSO group compared with the control group, the DSS + AG groups (75 mg/kg and 100 mg/kg), and the DSS + 5-ASA group (*P* < 0.05, Figures 1D,E).

Compared with that in the mice in the DSS + PBS group and the DSS + DMSO group, the colon damage in the mice in the groups treated with AG or 5-ASA was significantly reduced, including improved histological structure, reduced epithelial disintegration, and reduced inflammatory cell infiltration (Figures 1F,G, Figure S1A).

### Effects of AG on Colon Pro-inflammatory Factors and the Colonic Infiltration of Inflammatory Cells in DSS-Treated Mice

**Figure 2:**
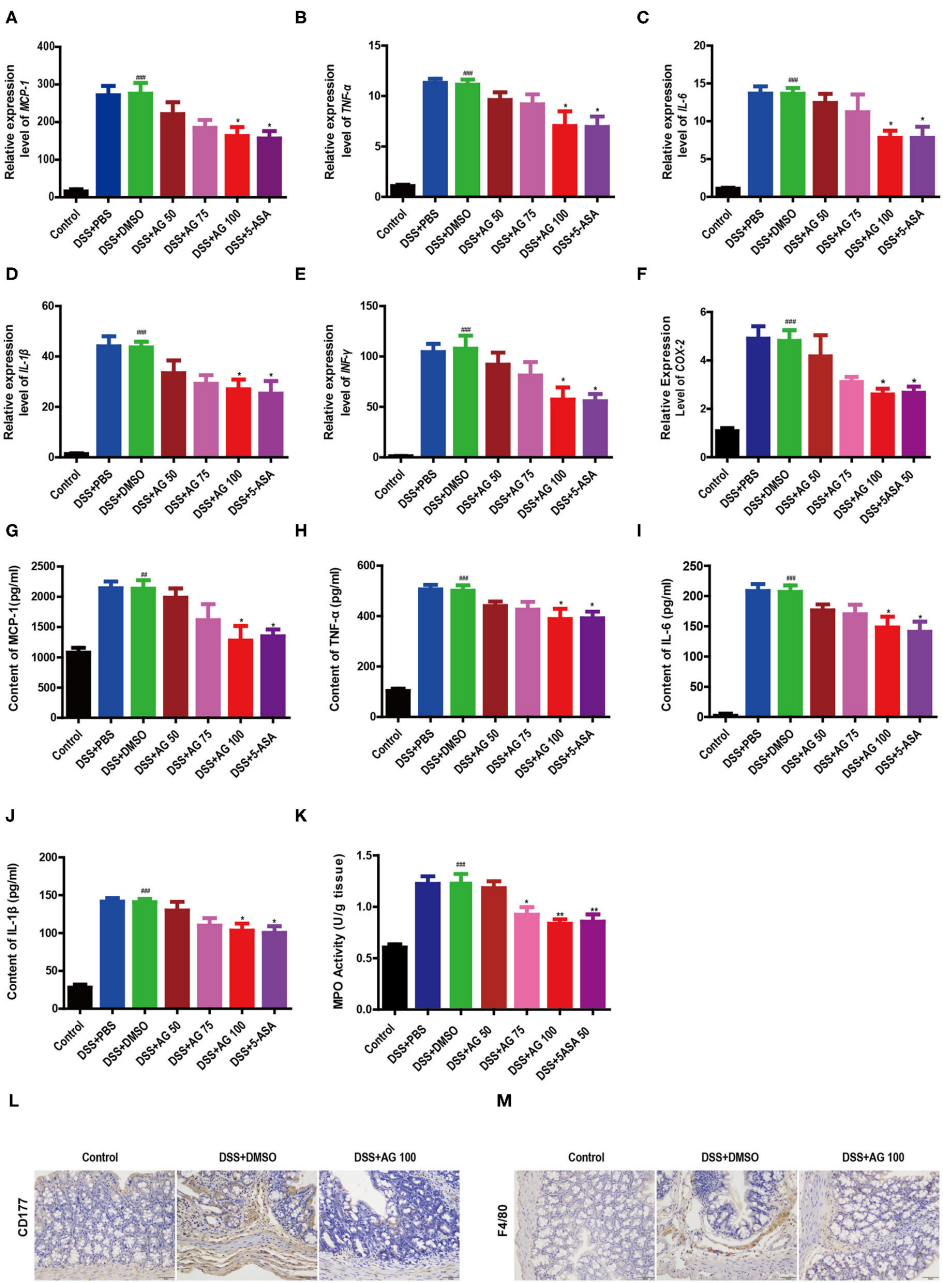
Effects of AG on colon cytokines and the colonic infiltration of inflammatory cells in DSS-treated mice. Expression levels of **(A)** *MCP-1*, **(B)** *TNF-*α, **(C)** *IL-6*, **(D)** *IL-1*β, **(E)** *IFN*γ, and **(F)** *COX-2* in the colon of acute colitis model mice determined by RT-qPCR. Colon tissue pro-inflammatory **(G)** MCP-1, **(H)** TNF-α, **(I)** L-6, **(J)**, and IL-1β levels. **(K)** MPO activity in the colon measured with an MPO assay kit. Data are expressed as the mean ± SEM, *n* = 8. Data were analyzed using one-way ANOVA (*post hoc* Tukey's multiple-comparison test) ^*##*^*P* < 0.01 and ^###^*P* < 0.001 compared with the control group. *P < 0.05 and **P < 0.01 compared with the DSS + DMSO group. Representative immunostaining images of colon sections stained for **(L)** CD177 and **(M)** F4/80. Formalin-fixed, paraffin-embedded 3 μm cross-sections were stained with the appropriate primary antibody. Scale bar: 50 μm.

Compared with those in the control group, the relative mRNA expression levels of pro-inflammatory factors, such as *MCP-1, TNF-*α, *IL-6, IL-1*β, IFN-γ, and cyclooxygenase-2 (*COX-2*), in the DSS-treated group were significantly enhanced (Figures 2A–F). Similar to 5-ASA, AG (100 mg/kg·BW) significantly suppressed the relative mRNA expression levels of these factors. Compared with those in the control group, the levels of all pro-inflammatory cytokines (MCP-1, TNF-α, IL-1β, and IL-6) in the DSS + DMSO group were increased significantly (Figures 2G–J). After treatment with 100 mg/kg·BW AG, the levels of pro-inflammatory cytokines were reduced significantly.

The results further showed that the MPO activity in the DSS + DMSO group was increased significantly (Figure 2K). Compared with that in the DSS + DMSO group, the content of MPO in the DSS + AG50 group (50 mg/kg·BW) presented no obvious change, but the DSS + AG groups (75 and 100 mg/kg·BW) and the DSS + 5-ASA group generally showed an attenuation of the increasing MPO activity. Targeting MPO may mitigate oxidative damage to host tissue and ensuing inflammation.

Compared with control treatment, DSS caused increased infiltration of CD177^+^ neutrophils (Figure 2L and Figure S1B) and F4/80^+^ macrophages (Figure 2M and Figure S1C). The DSS + AG 100 and DSS + 5-ASA groups showed reduced infiltration of CD177^+^ neutrophils and F4/80^+^ macrophages. In summary, AG treatment may inhibit the infiltration of neutrophils and macrophages into the colonic lesional area, thereby reducing the severity of complications caused by UC.

### AG Ameliorated Metabolic Endotoxemia and Improved Intestinal Mucosal Barrier Function

**Figure 3:**
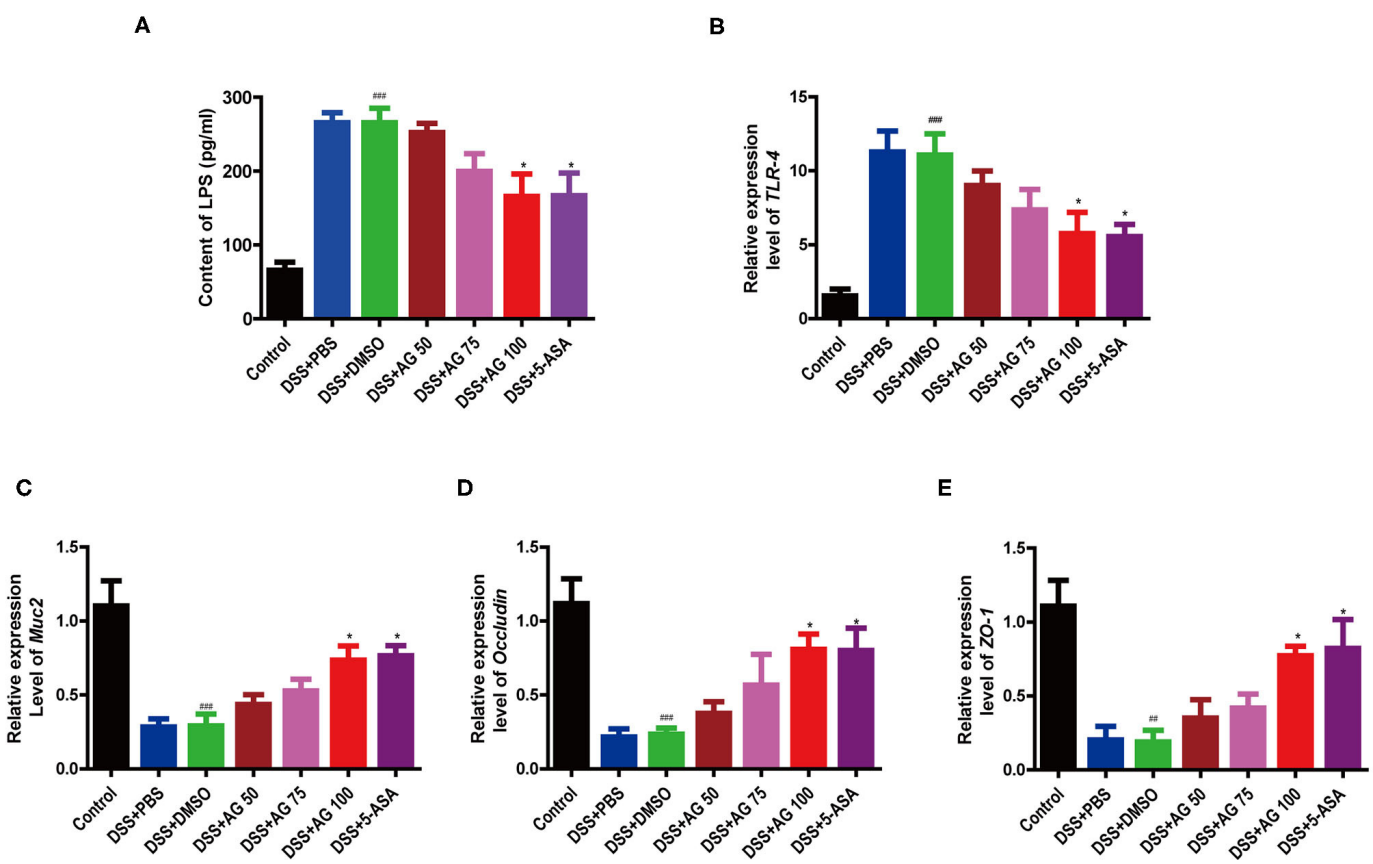
Effects of AG on metabolic endotoxemia and intestinal mucosal barrier function. Blood serum LPS level **(A)**. Relative expression levels of **(B)** *TLR4*, **(C)** *Muc2*, **(D)** *Occludin, and* **(E)** *ZO-1* in the colon. Data were analyzed using one-way ANOVA (*post hoc* Tukey's multiple-comparison test). ^###^*P* < 0.001 compared with the control group. *P < 0.05 compared with the DSS + DMSO group.

Compared with those in the control group, the mice in the DSS + PBS group and the DSS + DMSO group had significantly higher blood serum levels of lipopolysaccharide (LPS), indicating that DSS triggered metabolic endotoxemia. In the AG treatment groups, the LPS levels were lower than those in the DSS + PBS group and the DSS + DMSO group (Figure 3A) and close to those in the control group. AG (100 mg/kg·BW) significantly decreased the LPS content in the serum and ameliorated endotoxemia.

Compared with DSS + DMSO, AG significantly suppressed the relative mRNA expression levels of Toll-like receptor 4 (*TLR4*) and elevated the mRNA expression of *ZO-1, Occludin*, and *Muc2* (Figures 3B–E). In general, AG regulated the expression of these genes, maintaining them at levels close to those in the control group, and effectively alleviated DSS-induced metabolic endotoxemia and intestinal barrier damage.

### AG Inhibited NF-κB Pathway Activation

**Figure 4:**
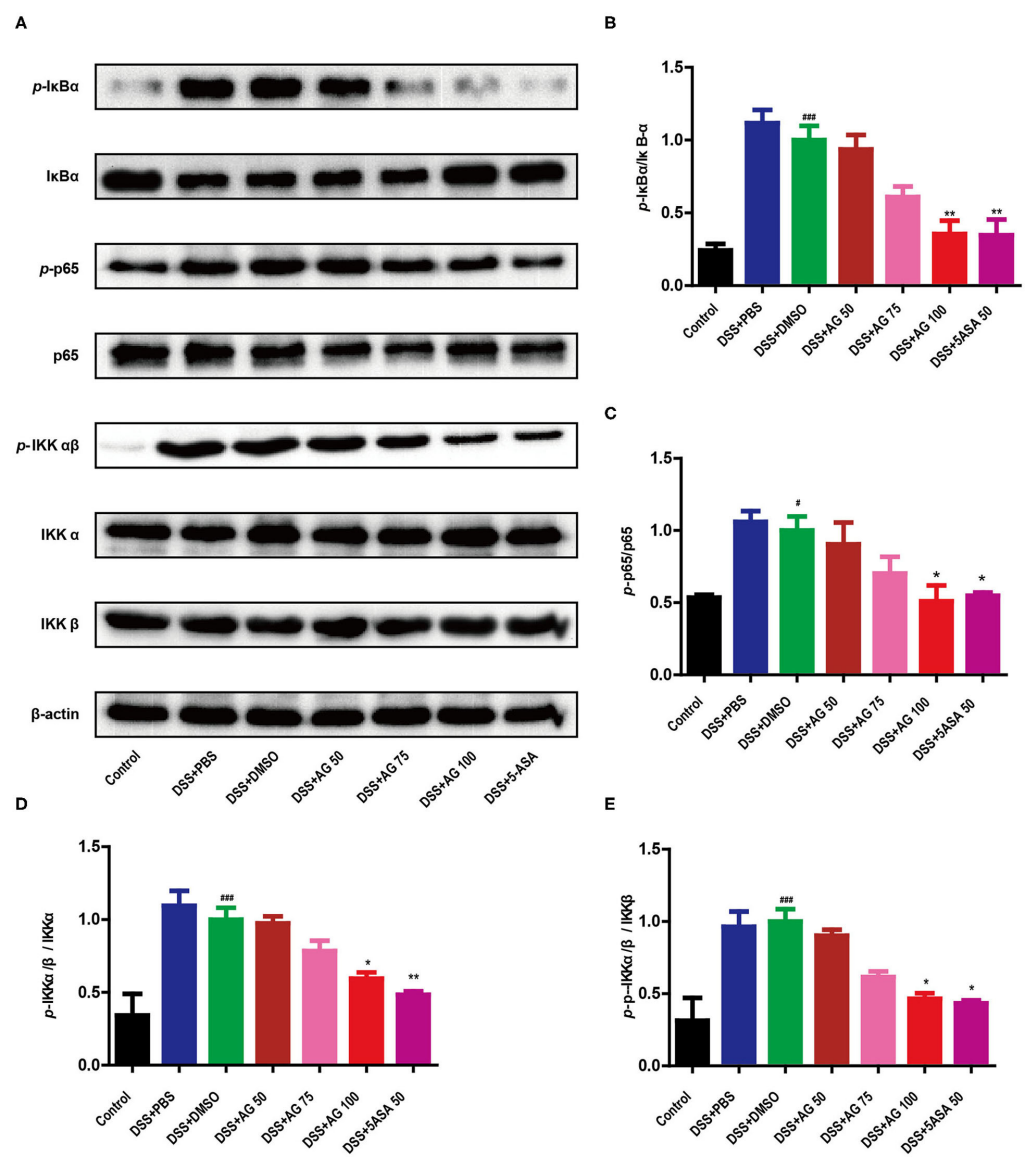
AG suppressed the NF-κB signaling pathway. The phosphorylation of IκBα, p65, and IKKα/β was detected by Western blotting **(A)**. **(B–E)** The relative density of each signaling band was calculated. β-actin was used as the protein loading control. Data were obtained from three independent experiments and are presented as the mean ± SEM. Data were analyzed using one-way ANOVA (*post hoc* Tukey's multiple-comparison test). *P < 0.05 and ^###^*P* < 0.001 compared with the control group. *P < 0.05 and **P < 0.01 compared with the DSS + DMSO group.

As shown in Figure 4, compared with control treatment, DSS significantly upregulated the expression of p-IκBα, p-IKKα/β, and p-p65 in the colon of the mice in the DSS + PBS group and the DSS + DMSO group (*P* < 0.05). Similar to 5-ASA, AG (100 mg/kg·BW) significantly downregulated the expression of p-IκBα, p-IKKα/β, and p-p65 compared with DSS + DMSO (*P* < 0.05, Figures 4A–D). The ratio of target phosphorylated protein expression to corresponding total protein expression was calculated as the protein expression level (Figures 4B–E). In summary, AG suppressed the production of pro-inflammatory cytokines by blocking the NF-κB pathway.

### AG Altered the Gut Microbiota in Colitis Mice

The V3-V4 regions of the 16S rRNA gene were sequenced to evaluate the effects of AG (100 mg/kg·BW) on DSS-induced UC mice. We obtained 1,026,250 sequences from all 18 samples from the control, DSS + DMSO and DSS + AG100 groups, each with more than 30,379 valid sequences for subsequent taxonomic analysis.

**Figure 5:**
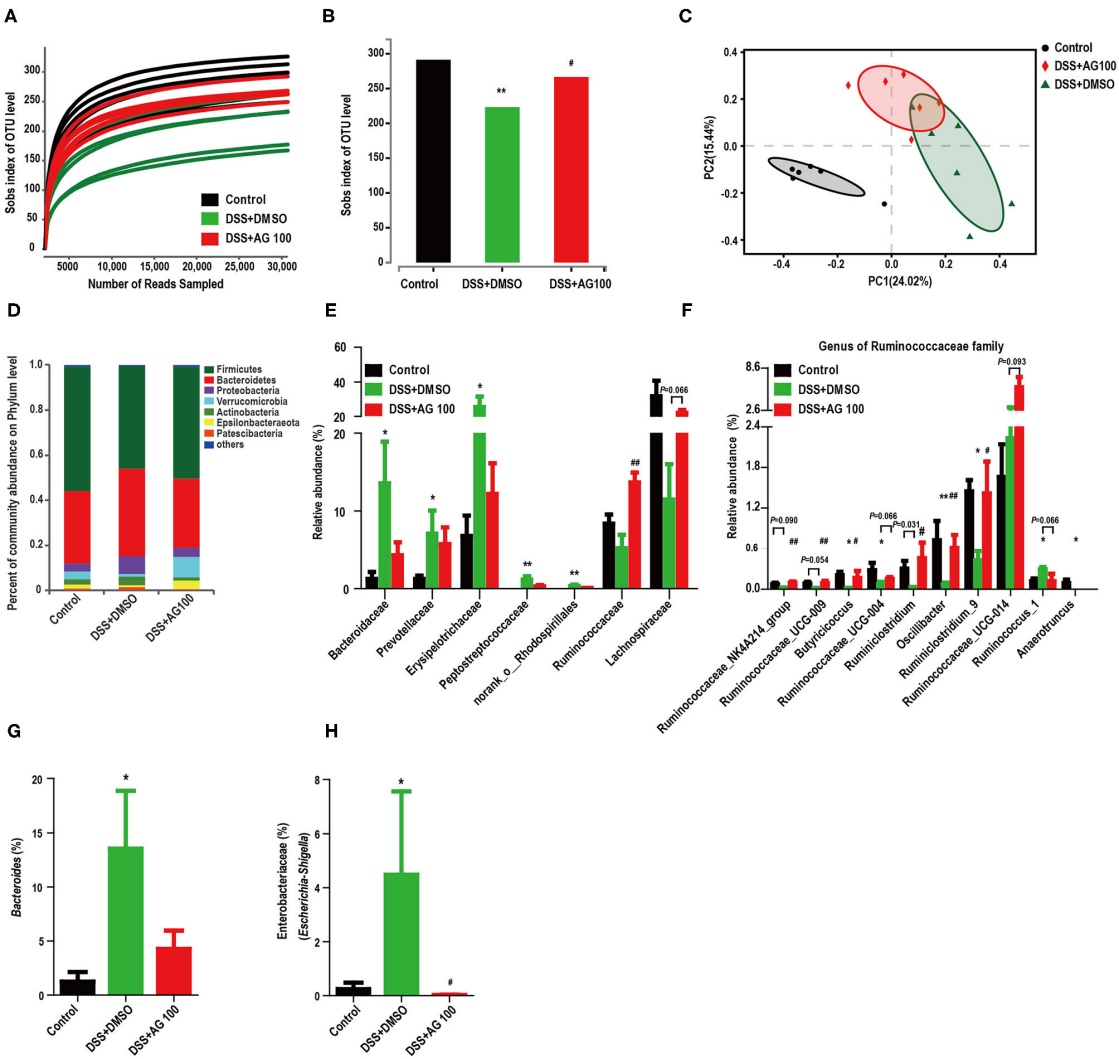
AG altered the diversity and composition of the gut microbiota in colitis mice. **(A)** The rarefaction curve of the Sobs index of each sample plateau. **(B)** Alpha diversity estimated by the Sobs index. **(C)** PCoA plot based on the Bray-Curtis index. **(D)** Relative abundances of predominant gut microbiota at the phylum level. **(E)** Relative abundances of the gut microbial community at the family level. **(F)** Relative abundances of genera in the Ruminococcaceae family. **(G)** Relative abundances of *Bacteroides* species. **(H)** Relative abundances of Enterobacteriaceae (*Escherichia-Shigella*). * or ^#^ indicates a significant difference between two groups using an unpaired two-tailed Student's *t*-test (alpha diversity) or the Wilcoxon rank-sum test (composition of the gut microbiota). *P < 0.05 and **P < 0.01 compared with the control group. ^#^*P* < 0.05 and ^##^*P* < 0.01 compared with the DSS + DMSO group.

The rarefaction curve of the Sobs index of each sample plateau (Figure 5A) with the current sequencing indicates that the sequencing depth was sufficient to reflect the diversity of the sample, and the sequencing result was credible. The alpha diversity of a sample reflects the richness and diversity of the microbial community. The community richness (Sobs, Chao and Ace), community diversity (Shannon) and community evenness (Shannoneven) indices were all significantly reduced in the DSS + DMSO group compared with the control group (Figure 5B and Figures S2A–S2D, *P* < 0.05). AG (100 mg/kg·BW) administration reversed these DSS-induced diversity index changes to varying degrees. Notably, AG (100 mg/kg·BW) administration significantly improved the DSS-induced decrease in the Sobs index (*P* < 0.05, Figure 5B). These results indicated that AG treatment could increase the gut microbiota diversity of DSS-induced UC mice. The beta diversity is displayed in Figure 5C. Principal coordinate analysis (PCoA) results showed that DSS changed the gut microbial structure significantly. Although the administration of high-dose AG (100 mg/kg·BW) could not completely reverse the effect on the gut microbiota, AG still appeared to regulate the abnormal gut microbiota in DSS-induced UC mice.

Histograms were used to indicate the relative abundances of gut microbial species in different groups. Compared with control treatment, DSS treatment increased the relative abundance of Proteobacteria and decreased the relative abundance of Verrucomicrobia, whereas AG treatment reversed these changes (Figure 5D); however, the differences were not significant (Figure 5D, Figures S2E,S2F). Figure S2G and Figure 5E show the changes in the main microbiota at the family level. The relative abundances of Prevotellaceae, Erysipelotrichaceae, Bacteroidaceae, Peptostreptococcaceae, and norank_o__Rhodospirillales were significantly increased in the DSS + DMSO group compared with the control group, whereas AG treatment reversed these changes to various degrees. In contrast, DSS decreased the relative abundances of the Ruminococcaceae and Lachnospiraceae families, while AG treatment reversed these changes. Compared with control treatment, DSS treatment significantly reduced the relative abundances of most genera belonging to the Ruminococcaceae family, whereas AG treatment significantly reversed most of these changes (Figure 5F). Notably, DSS treatment significantly increased the relative abundance of the genus *Ruminococcus_1*, whereas AG treatment reduced the relative abundance to a level close to that seen in the control group. Most genera belonging to the Lachnospiraceae family showed similar patterns as those in the Ruminococcaceae family (Figure S2H). Notably, the relative abundance of LPS-producing bacteria (*Bacteroides* and *Escherichia-Shigella*) was significantly increased in DSS-induced colitis mice, while AG inhibited this effect (Figures 5G,H).

**Figure 6:**
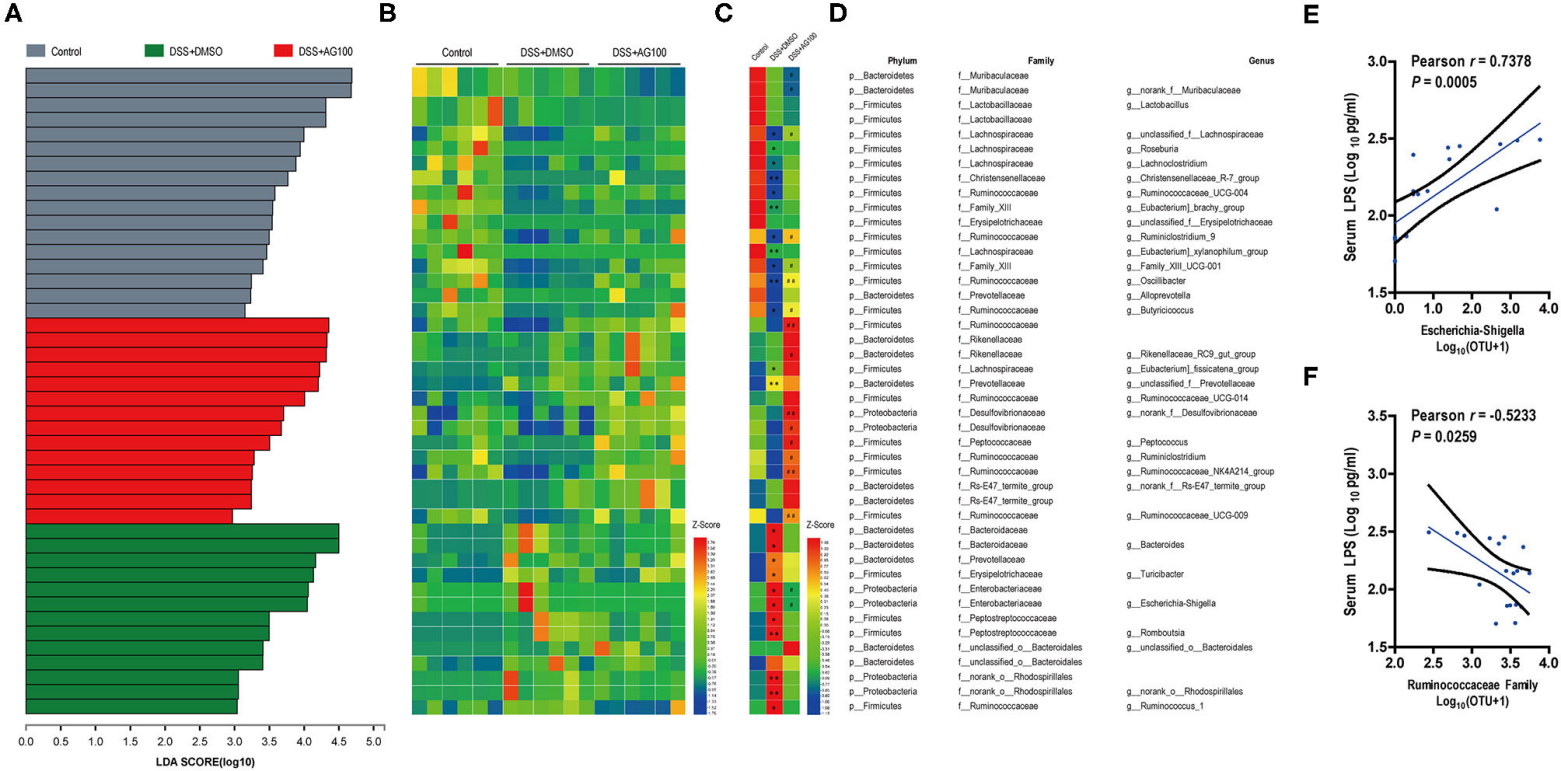
Gut microbiota composition and its correlation with metabolic endotoxemia. **(A)** Linear discriminant analysis (LDA) at the family and genus levels. **(B)** Heatmap showing the relative abundances of different genera and families. **(C)** Heatmap showing the changing directions of genera and families in different groups. The asterisk (*) indicates the genus or family in the control group altered by DSS. The hash tag (^#^) indicates the genus or family in the DSS + DMSO group altered by AG 100. **(D)** Representative bacterial taxa information (phylum, family, and genus). The Wilcoxon rank-sum test was used to calculate differences in the relative abundances of genera or families. A *P* < 0.05 was considered significant. *P < 0.05 and **P < 0.01 compared with the control group. ^#^*P* < 0.05 and ^##^*P* < 0.01 compared with the DSS + DMSO group. **(E)** Correlation between *Escherichia-Shigella* and LPS determined by Pearson's correlation analysis. **(F)** Correlation between the Ruminococcaceae family and LPS determined by Pearson's correlation analysis.

Linear discriminant analysis effect size (LEfSe) analyses were used to explore which bacterium might be responsible for the impact on DSS-induced UC mice. The dominant microbiota at the family and genus levels for each group (Figure S2I and Figure 6A) were obtained. Then, a heatmap was constructed to display the 44 dominant microbiota in the 3 groups (Figures 6B–D). Specifically, 24 distinct families or genera were significantly reversed by AG treatment. Muribaculaceae, Ruminococcaceae, and Bacteroidaceae had the highest linear discriminant analysis (LDA) scores in the control group, the DSS+DMSO group, and the DSS+AG100 group, respectively, at the family level. Then, the correlations between Ruminococcaceae, *Escherichia-Shigella* and LPS were analyzed by Pearson's correlation analysis (Figures 6E,F). *Escherichia-Shigella* and LPS showed a significant positive correlation (Pearson *r* = 0.7378, *P* < 0.001). In contrast, Ruminococcaceae and LPS were negatively correlated (Pearson *r* = −0.5233, *P* < 0.05).

## Discussion

UC is well-known due to its high prevalence worldwide. In this study, a DSS-induced mouse model was used to simulate clinical UC. After a 7-day induction with 3% DSS, the watery feces and decreasing body weights of the mice in the DSS + PBS and DSS + DMSO groups indicated successful model establishment. A series of studies have shown that flavonoids and their derivatives have good ameliorative effects on UC (18–20). We conducted the first study on the improvement of colitis by AG, a natural flavonoid that is widely found in various medicinal and edible plants. Our results demonstrated that AG treatment could attenuate UC, as evidenced by the decreases in weight loss, colonic shortening, DAI scores, and histological scores in the AG groups compared with the DSS group, and 100 mg/kg·BW AG had the best effect (Figure 1).

Reductions in inflammatory cytokine levels in the serum represent a logical goal for IBD therapy (21). The dramatically elevated mRNA expression levels of *MCP-1, TNF-*α, *IL-6, IL-1*β, and *IFN-*γ in the colon are important features of the DSS colitis model (21, 22). In the present study, 100 mg/kg·BW AG significantly reduced the DSS-induced increases in the relative mRNA expression levels of *MCP-1, TNF-*α, *IL-6, IL-1*β, and *IFN-*γ in the colon and the protein expression levels of pro-inflammatory factors in the colon, including MCP-1, TNF-α, IL-6, and IL-1β. The increased levels of pro-inflammatory cytokines in the colon of DSS-treated mice are usually caused by the infiltration of inflammatory cells. In the present study, increased colonic infiltration by macrophages and neutrophils was observed in DSS-induced colitis; AG treatment also improved this phenomenon (Figure 2).

Lipopolysaccharide (LPS) is a cell-associated glycolipid that makes up the outer leaflet of the outer membrane of Gram-negative bacteria (23). LPS can promote the expression of pro-inflammatory cytokines and activate innate immune cells, such as macrophages, by stimulating signaling through TLR4 (24, 25). Consistent with the effect of AG on the DSS-induced expression of pro-inflammatory cytokines in the colon, 100 mg/kg·BW AG significantly reduced the increased LPS content in the serum, F4/80^+^ macrophage infiltration and relative mRNA expression levels of *TLR4* in colon tissues induced by DSS treatment in this study.

The intestinal epithelium, which is covered with a mucus gel layer, separates the luminal microbiota from systemic tissue (26). From the results for distal colonic sections, the mucus gel layer was completely damaged in the DSS group. *Muc2* represents the main component of the colonic mucus. Deletion of Muc2 in mice eliminates mucus, causing bacterial colonization of the crypts and inflammation (26). DSS treatment significantly reduced *Muc2* mRNA expression to a low level, while AG restored this expression to the level seen in the control group in the present study (Figure 3).

Maintaining the tightness and integrity of the intestinal barrier is also an important goal in the treatment of UC (21). The mRNA expression of intestinal tight junction-associated proteins (*ZO-1* and *Occludin*) also suggested significant ameliorative effects for AG (Figure 3).

NF-κB is a key regulator of inflammation, innate immunity, and tissue integrity (27). The nuclear translocation of NF-κB is strongly activated in experimental colitis model animals and IBD patients (28). In the present study, the NF-κB signaling pathway was significantly activated in DSS-induced colitis mice.

Previous studies have shown that AG can inhibit the expression of lipopolysaccharide-induced inflammatory mediators in macrophages through the NF-κB pathway; AG can reduce mastitis (29), lung injury (30), uterine and epithelial inflammation (31), and colitis (32) in mice or rats by regulating the expression of NF-κB signaling components. Consistent with previous studies, this study showed that AG suppressed the pro-inflammatory response by inhibiting the activation of the NF-κB pathway (Figure 4).

UC was previously thought to be an autoimmune disease but is now considered an infectious disease of the gut microbiota (33). In previous studies, DSS-treated mice were shown to exhibit dysbiosis of the gut microbiota, reflected as reductions in the numbers of symbionts and commensalists and/or an increase in the numbers of pathobionts. Reduced microbial diversity is a common phenomenon in UC (33). Herein, the microbiota community composition in the DSS-treated groups was significantly different from that in the control group. AG treatment improved the alpha diversity and changed the community composition.

At the phylum level, AG treatment reversed the DSS-induced changes in the relative abundances of Proteobacteria and Verrucomicrobia. Most Proteobacteria are thought to be harmful bacteria (33). Enterobacteriaceae (*Escherichia-Shigella*) is the typical family (genus) of Proteobacteria and includes LPS-producing, Gram-negative bacteria. Its relative abundance in the cecal content of DSS-induced colitis mice was increased more than 10 times than that in the cecal content of the mice in the control group; AG treatment significantly reduced its abundance to a level lower than that seen in the control group. It is worth noting that *Escherichia-Shigella* and LPS showed a significant positive correlation. This finding suggests that the ameliorative effect of AG on DSS-induced colitis is partly achieved by reducing the abundance of the classic pathogen genus *Escherichia-Shigella*. To date, *Akkermansia* is the only genus of bacteria belonging to Verrucomicrobia known to be in the gut. Emerging evidence has shown that reduced levels of *Akkermansia* are observed in IBD patients with inflammatory bowel diseases and metabolic disorders (34). In this study, AG reversed the reduction in Verrucomicrobia abundance in DSS-induced colitis mice.

At the family level, DSS treatment significantly increased the relative abundances of Bacteroidaceae, Prevotellaceae, Erysipelotrichaceae, Peptostreptococcaceae, and norank_o__Rhodospirillales and decreased the relative abundances of Ruminococcaceae and Lachnospiraceae, whereas AG treatment reversed these changes to varying degrees. In previous studies on UC, it was also shown that the abundances of colitogenic strains (Bacteroidaceae and Erysipelotrichaceae) are increased by induction with DSS (33, 35, 36) and that their abundances are relatively high in patients with IBD (37, 38). Notably, most Bacteroidaceae and Erysipelotrichaceae bacteria have the ability to produce LPS. Ruminococcaceae and Lachnospiraceae have been recognized as probiotics that can produce SCFAs, such as *n*-butyric acid. DSS-induced UC animals (39, 40) and IBD patients (41, 42) always have lower abundances of Ruminococcaceae and Lachnospiraceae than healthy controls. Notably, Ruminococcaceae and LPS showed a significant negative correlation. Interestingly but puzzlingly, DSS increased the abundances of Prevotellaceae and Peptostreptococcaceae, which are thought to be potentially beneficial families.

At the genus level (Figure 6), *Family_XIII_UCG-001, Unclassified _f__ Lachnospiraceae, Ruminiclostridium_9, Oscillibacter, Butyricicoccus, Ruminiclostridium, Ruminococcaceae_NK4A214_group, Ruminococcaceae_ UCG-009*, and *Peptococcus* showed low relative abundances in the DSS + DMSO group, patterns that were significantly reversed by AG (*P* < 0.05). Among these genera, *Ruminiclostridium_9, Oscillibacter, Butyricicoccus, Ruminiclostridium, Ruminococcaceae_NK4A214_group*, and *Ruminococcaceae_UCG-009* all belong to the Ruminococcaceae family. These reductions are characteristics of the gut microbiota in DSS-treated mice that were observed in previous studies (43–45).

Notably, *Oscillibacter* is associated with severe DSS-induced colitis (21, 46). *B. pullicaecorum* (a species of *Butyricicoccus*) could attenuate TNBS-induced colitis in rats, and a supernatant derived from a *B. pullicaecorum* culture could strengthen epithelial barrier function. Our results demonstrated that the ameliorative effects of AG on DSS-induced UC symptoms were achieved by changing the gut microbiota, especially certain specific microbiomes, and we concluded that Ruminococcaceae and Enterobacteriaceae (*Escherichia*-*Shigella*) might be the key groups affected by AG to improve UC.

**Figure 7:**
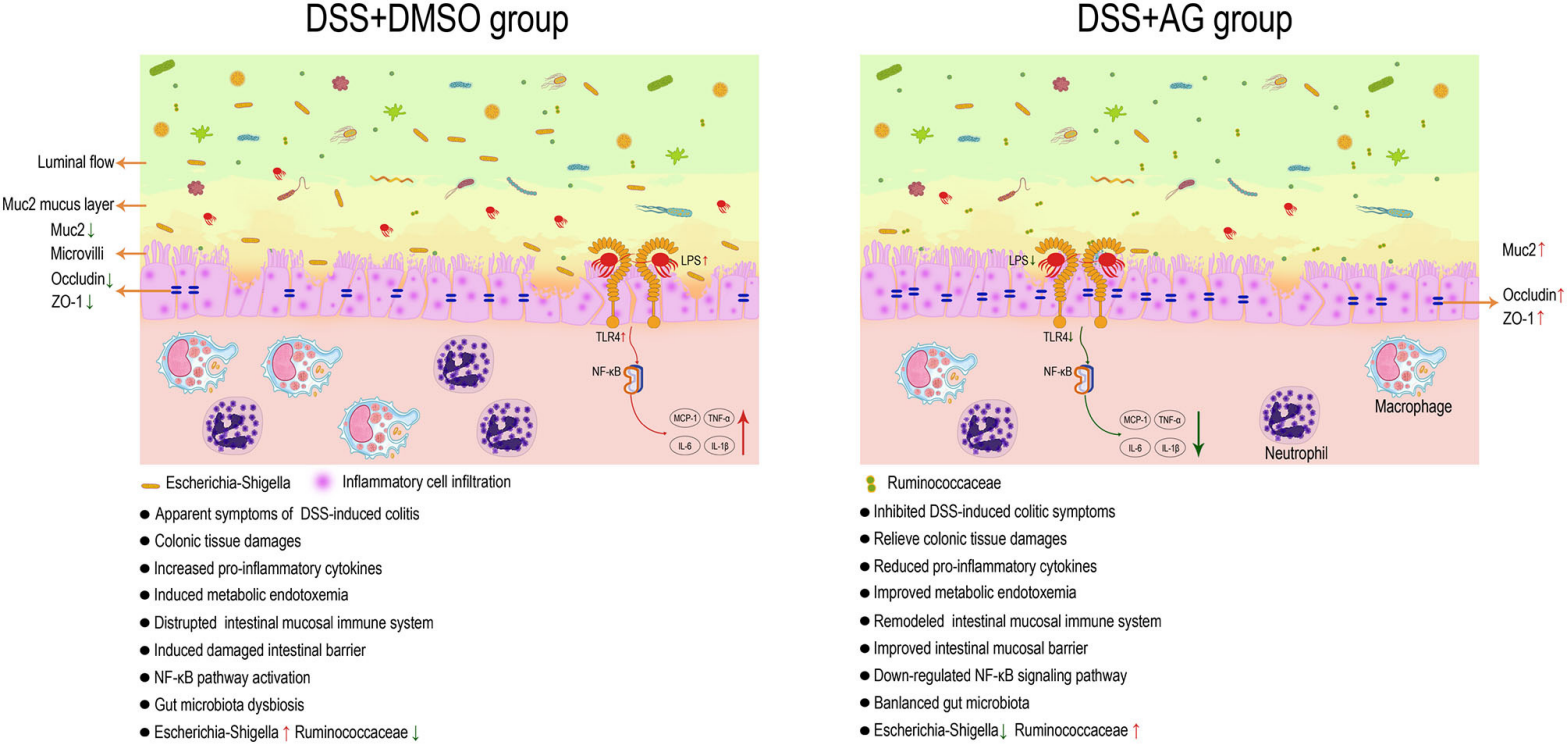
AG exerts a good anti-UC effect through microbiota/LPS/TLR4/NF-κB-related pathways in mice. Astragalin (AG) attenuated the symptoms of DSS-induced ulcerative colitis (UC) in mice. AG inhibited the activation of pro-inflammatory cytokines and the NF-κB signaling pathway, improved gut barrier function and metabolic endotoxemia, and partially reversed the alteration in the gut microbiota in UC mice. Ruminococcaceae and *Escherichia-Shigella* were thought to be the key groups affected by AG to improve UC.

In conclusion, AG exerted protective effects against UC through several targets, including metabolic endotoxemia (serum LPS), pro-inflammatory cytokines (COX-2, MCP-1, TNF-α, IL-6, IL-1β, and IFN-γ), enhancement of the mRNA expression levels of mucin (*Muc2*) and tight junction proteins (*ZO-1* and *occludin*) in the colon, inhibition of the relative mRNA expression levels of *TLR4*, inhibition of NF-κB pathway activation and modulation of the gut microbiota. Through systematic data analysis and literature review, key bacteria related to UC were identified (Ruminococcaceae and *Escherichia-Shigella*). In summary, AG might exert good anti-inflammatory effects by affecting the microbiota/LPS/TLR4/NF-κB-related pathway (Figure 7).

## Data Availability Statement

The datasets generated for this study can be found in online repositories. The names of the repository/repositories and accession number(s) can be found at: https://www.ncbi.nlm.nih.gov/genbank/, PRJNA578013.

## Ethics Statement

The animal study was reviewed and approved by Yunnan Agricultural University Animal Ethics Committee with respect to ethical issues and scientific care (YNAU-2018-031).

## Author Contributions

YT and JS designed the experiments. LP, XG, and LN performed the animal studies. LP, XG, LN, YW, and JX performed the molecular biology experiments. XG, LP, CS, and LT analyzed the 16S rRNA gene sequencing data. LP, XG, YT, and JS prepared the manuscript and had primary responsibility for the final content. All authors read and approved the final manuscript.

## Conflict of Interest

The authors declare that the research was conducted in the absence of any commercial or financial relationships that could be construed as a potential conflict of interest.
