# Supplementary material for: Astragalin Attenuates Dextran Sulfate Sodium (DSS)-Induced Acute Experimental Colitis by Alleviating Gut Microbiota Dysbiosis and Inhibiting NF-κB Activation in Mice
Source: Front Immunol. 2020 Sep 15;11:2058. doi: 10.3389/fimmu.2020.02058 (PMC7523281; doi:10.3389/fimmu.2020.02058)
Supplement: Supplementary file 1 [file Presentation_1.PPT]

## Slide 1
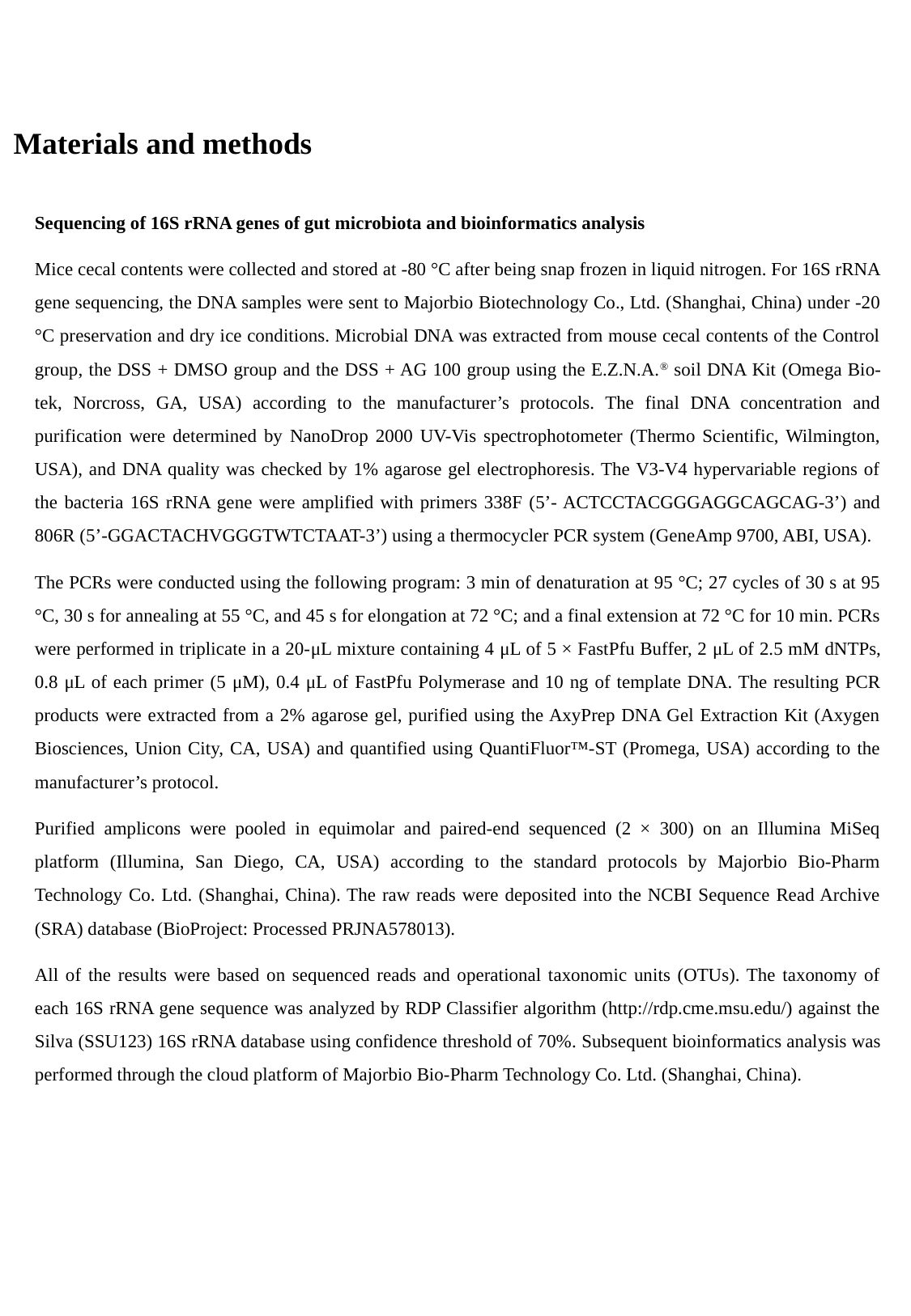

Materials and methods
Sequencing of 16S rRNA genes of gut microbiota and bioinformatics analysis
Mice cecal contents were collected and stored at -80 °C after being snap frozen in liquid nitrogen. For 16S rRNA gene sequencing, the DNA samples were sent to Majorbio Biotechnology Co., Ltd. (Shanghai, China) under -20 °C preservation and dry ice conditions. Microbial DNA was extracted from mouse cecal contents of the Control group, the DSS + DMSO group and the DSS + AG 100 group using the E.Z.N.A.® soil DNA Kit (Omega Bio-tek, Norcross, GA, USA) according to the manufacturer’s protocols. The final DNA concentration and purification were determined by NanoDrop 2000 UV-Vis spectrophotometer (Thermo Scientific, Wilmington, USA), and DNA quality was checked by 1% agarose gel electrophoresis. The V3-V4 hypervariable regions of the bacteria 16S rRNA gene were amplified with primers 338F (5’- ACTCCTACGGGAGGCAGCAG-3’) and 806R (5’-GGACTACHVGGGTWTCTAAT-3’) using a thermocycler PCR system (GeneAmp 9700, ABI, USA).
The PCRs were conducted using the following program: 3 min of denaturation at 95 °C; 27 cycles of 30 s at 95 °C, 30 s for annealing at 55 °C, and 45 s for elongation at 72 °C; and a final extension at 72 °C for 10 min. PCRs were performed in triplicate in a 20-μL mixture containing 4 μL of 5 × FastPfu Buffer, 2 μL of 2.5 mM dNTPs, 0.8 μL of each primer (5 μM), 0.4 μL of FastPfu Polymerase and 10 ng of template DNA. The resulting PCR products were extracted from a 2% agarose gel, purified using the AxyPrep DNA Gel Extraction Kit (Axygen Biosciences, Union City, CA, USA) and quantified using QuantiFluor™-ST (Promega, USA) according to the manufacturer’s protocol.
Purified amplicons were pooled in equimolar and paired-end sequenced (2 × 300) on an Illumina MiSeq platform (Illumina, San Diego, CA, USA) according to the standard protocols by Majorbio Bio-Pharm Technology Co. Ltd. (Shanghai, China). The raw reads were deposited into the NCBI Sequence Read Archive (SRA) database (BioProject: Processed PRJNA578013).
All of the results were based on sequenced reads and operational taxonomic units (OTUs). The taxonomy of each 16S rRNA gene sequence was analyzed by RDP Classifier algorithm (http://rdp.cme.msu.edu/) against the Silva (SSU123) 16S rRNA database using confidence threshold of 70%. Subsequent bioinformatics analysis was performed through the cloud platform of Majorbio Bio-Pharm Technology Co. Ltd. (Shanghai, China).

## Slide 2
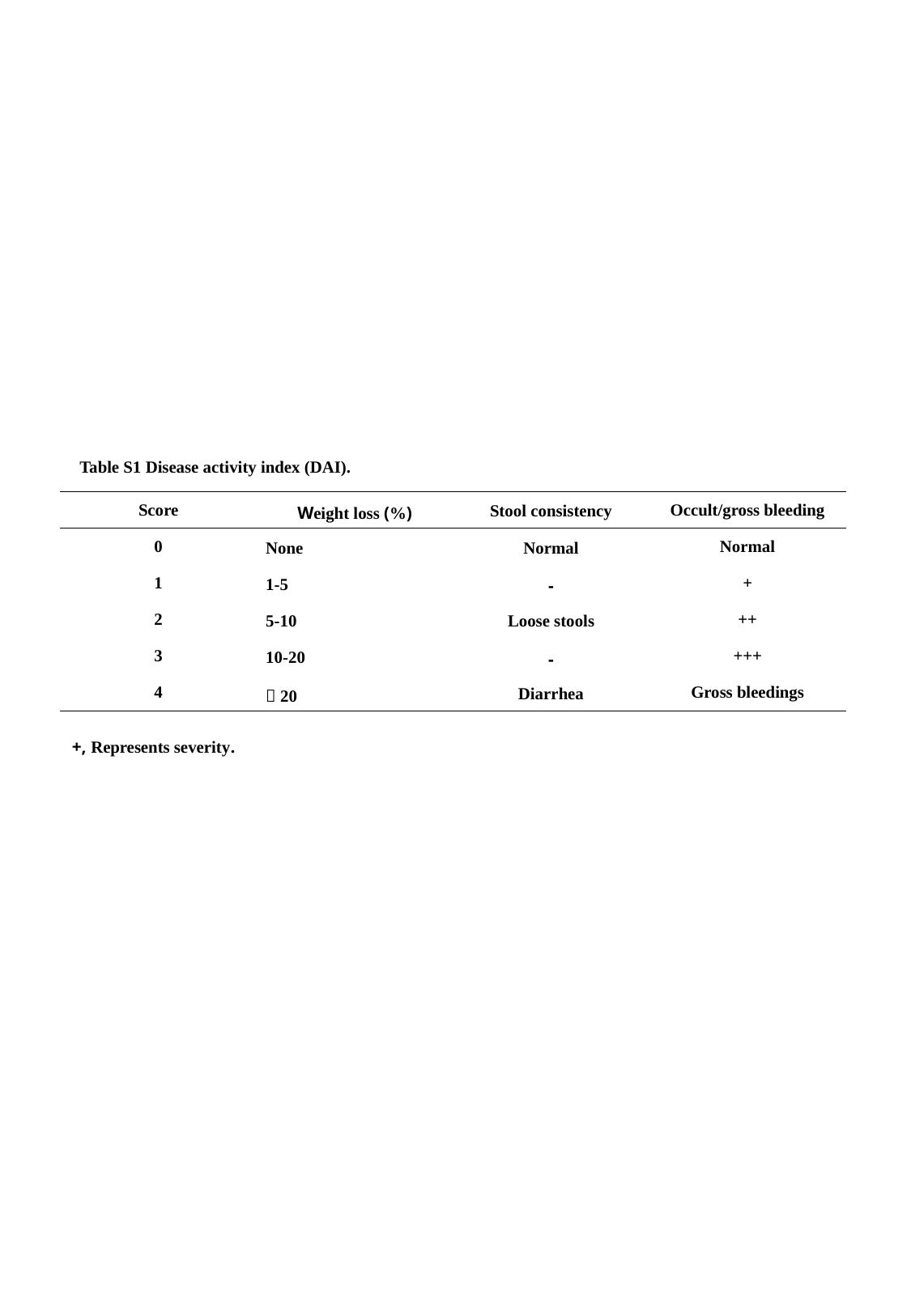

Table S1 Disease activity index (DAI).
| Score | Weight loss (%) | Stool consistency | Occult/gross bleeding |
| --- | --- | --- | --- |
| 0 | None | Normal | Normal |
| 1 | 1-5 | - | + |
| 2 | 5-10 | Loose stools | ++ |
| 3 | 10-20 | - | +++ |
| 4 | ＞20 | Diarrhea | Gross bleedings |
+, Represents severity.

## Slide 3
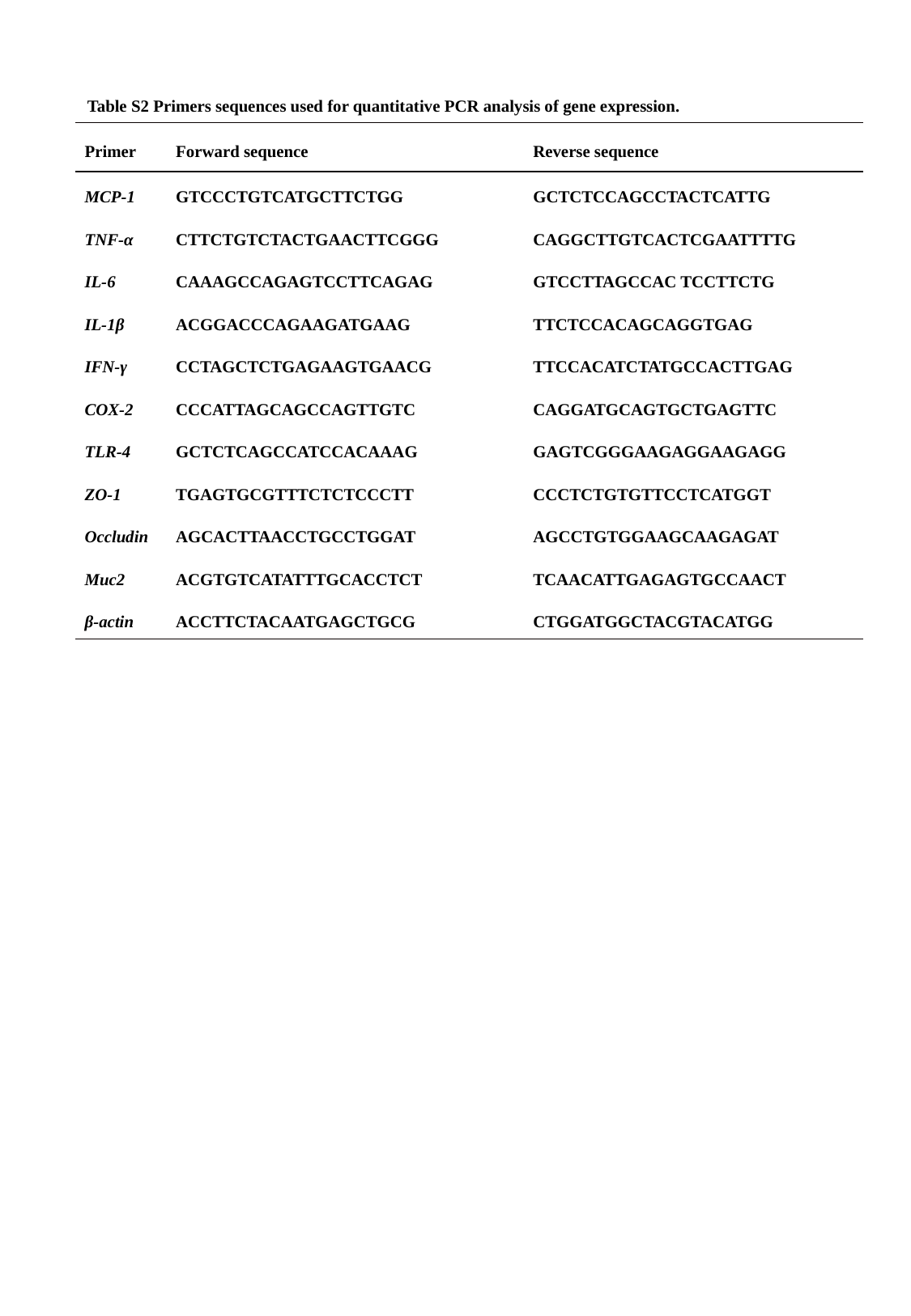

Table S2 Primers sequences used for quantitative PCR analysis of gene expression.
| Primer | Forward sequence | Reverse sequence |
| --- | --- | --- |
| MCP-1 | GTCCCTGTCATGCTTCTGG | GCTCTCCAGCCTACTCATTG |
| TNF-α | CTTCTGTCTACTGAACTTCGGG | CAGGCTTGTCACTCGAATTTTG |
| IL-6 | CAAAGCCAGAGTCCTTCAGAG | GTCCTTAGCCAC TCCTTCTG |
| IL-1β | ACGGACCCAGAAGATGAAG | TTCTCCACAGCAGGTGAG |
| IFN-γ | CCTAGCTCTGAGAAGTGAACG | TTCCACATCTATGCCACTTGAG |
| COX-2 | CCCATTAGCAGCCAGTTGTC | CAGGATGCAGTGCTGAGTTC |
| TLR-4 | GCTCTCAGCCATCCACAAAG | GAGTCGGGAAGAGGAAGAGG |
| ZO-1 | TGAGTGCGTTTCTCTCCCTT | CCCTCTGTGTTCCTCATGGT |
| Occludin | AGCACTTAACCTGCCTGGAT | AGCCTGTGGAAGCAAGAGAT |
| Muc2 | ACGTGTCATATTTGCACCTCT | TCAACATTGAGAGTGCCAACT |
| β-actin | ACCTTCTACAATGAGCTGCG | CTGGATGGCTACGTACATGG |

## Slide 4
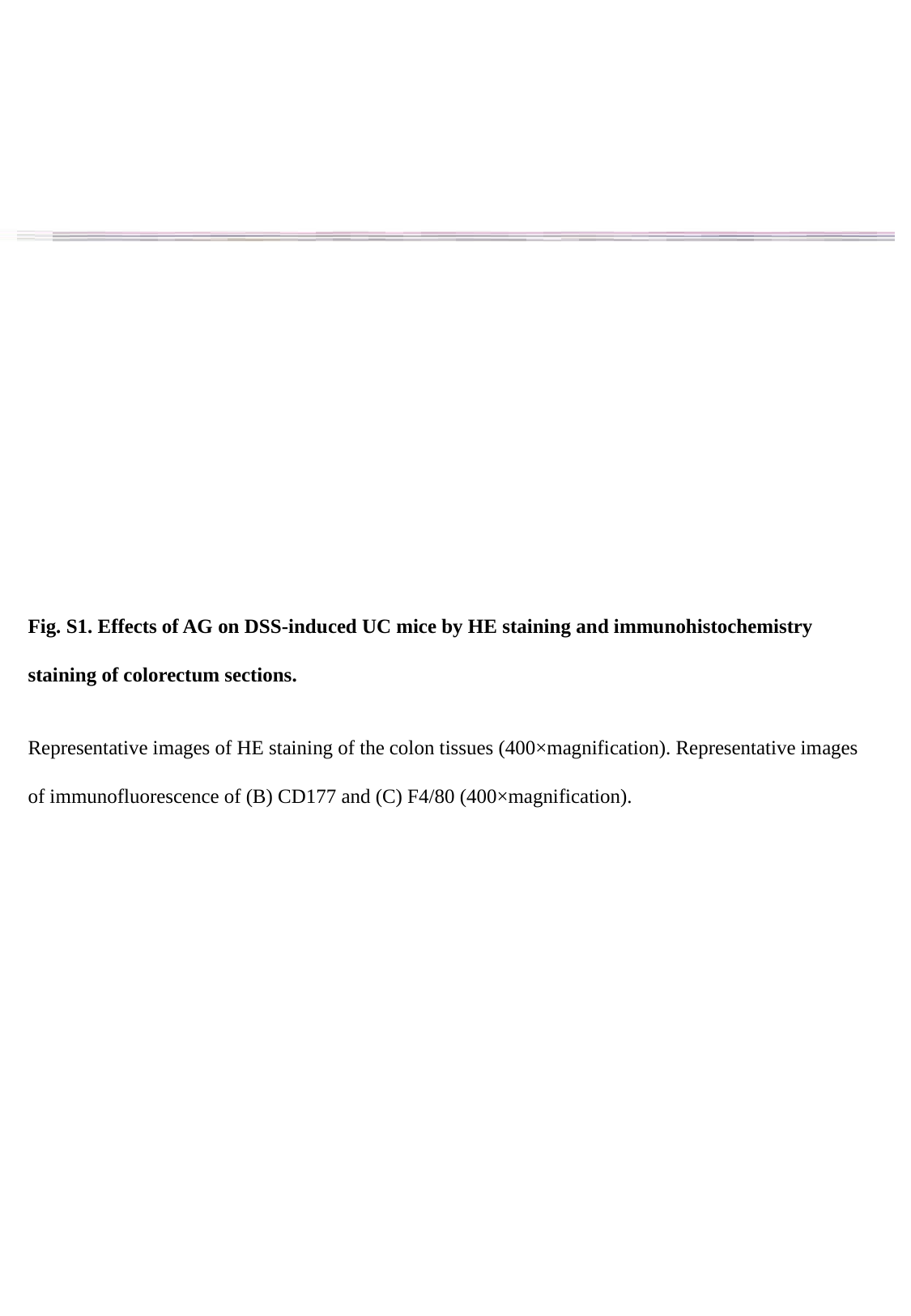

Fig. S1. Effects of AG on DSS-induced UC mice by HE staining and immunohistochemistry staining of colorectum sections.
Representative images of HE staining of the colon tissues (400×magnification). Representative images of immunofluorescence of (B) CD177 and (C) F4/80 (400×magnification).

## Slide 5
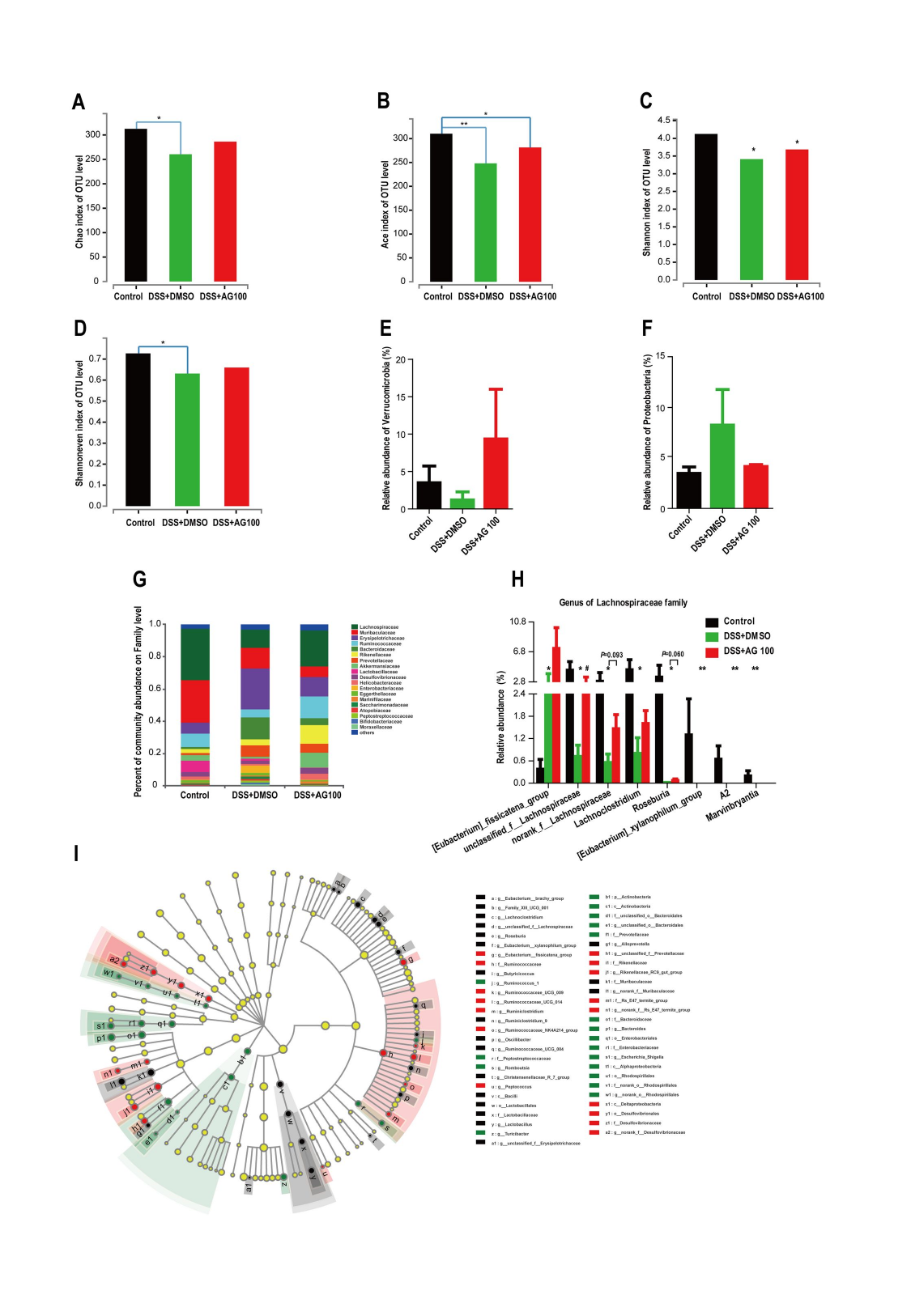

## Slide 6
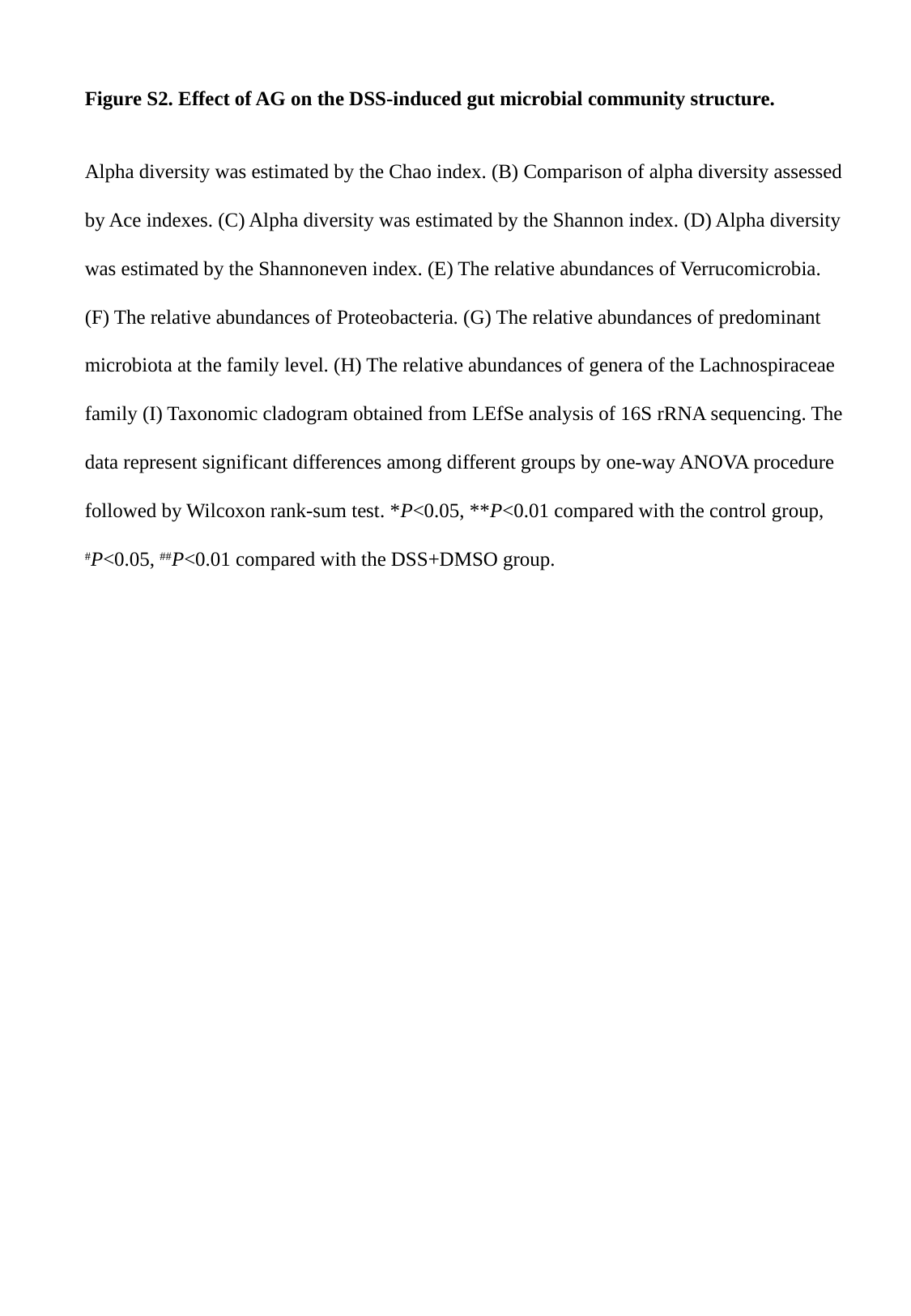

Figure S2. Effect of AG on the DSS-induced gut microbial community structure.
Alpha diversity was estimated by the Chao index. (B) Comparison of alpha diversity assessed by Ace indexes. (C) Alpha diversity was estimated by the Shannon index. (D) Alpha diversity was estimated by the Shannoneven index. (E) The relative abundances of Verrucomicrobia. (F) The relative abundances of Proteobacteria. (G) The relative abundances of predominant microbiota at the family level. (H) The relative abundances of genera of the Lachnospiraceae family (I) Taxonomic cladogram obtained from LEfSe analysis of 16S rRNA sequencing. The data represent significant differences among different groups by one-way ANOVA procedure followed by Wilcoxon rank-sum test. *P<0.05, **P<0.01 compared with the control group, #P<0.05, ##P<0.01 compared with the DSS+DMSO group.
